# A Case of Drug‐Resistant Renovascular Hypertension due to Renal Artery Stenosis Successfully Treated by Nephrectomy of the Affected Kidney

**DOI:** 10.1002/iju5.70084

**Published:** 2025-08-19

**Authors:** Moeto Shimoda, Shinji Otake, Masashi Imano, Tetsuya Aoki, Jurii Karibe, Yosuke Shibata, Masahiro Inoue, Akiko Nagatomo, Mayumi Yakeishi, Kazuki Kobayashi

**Affiliations:** ^1^ Department of Urology Yokosuka Kyosai Hospital Yokosuka Japan; ^2^ Department of Urology Yokohama City University Hospital Yokohama Japan; ^3^ Department of Nephrology Yokosuka Kyosai Hospital Yokosuka Japan; ^4^ Department of Pathology Yokosuka Kyosai Hospital Yokosuka Japan

**Keywords:** drug‐resistant hypertension, nephrectomy, percutaneous transluminal renal angioplasty, renal artery stenosis, renovascular hypertension

## Abstract

**Introduction:**

Renal artery stenosis (RAS) reduces renal blood flow and activates the renin‐angiotensin‐aldosterone (RAA) system, resulting in renovascular hypertension (RVH).

**Case Presentation:**

We report a case of a 49‐year‐old woman with RVH due to bilateral renal artery stenosis, predominantly on the right. Despite pharmacological treatment, blood pressure remained poorly controlled; leading to severe heart failure that required dialysis. Percutaneous renal angioplasty was considered but deemed technically difficult. The patient underwent a successful laparoscopic right nephrectomy, leading to improved blood pressure control and reduced need for antihypertensive medications.

**Summary**

- Nephrectomy may be an effective treatment option for renovascular RVH in cases where blood pressure is refractory to medical therapy, PTRA is not feasible, and the affected kidney is atrophic with impaired function.

## 1 Introduction

Renal artery stenosis (RAS) is a condition characterized by the narrowing of the renal artery lumen, leading to impaired renal blood flow [1]. The primary cause of RAS is atherosclerosis, which accounts for approximately 90% of cases. Other causes include fibromuscular dysplasia and vasculitis [2]. The mechanism of renovascular hypertension (RVH) caused by RAS involves activation of the renin‐angiotensin‐aldosterone (RAA) system, leading to worsening hypertension [3]. RVH is primarily managed with antihypertensive agents. However, in cases where blood pressure remains uncontrolled despite the use of these antihypertensive agents or when recurrent heart failure due to increased renin activity occurs, percutaneous transluminal renal angioplasty (PTRA) may be considered [4].

## 2 Case Presentation

**FIGURE 1:**
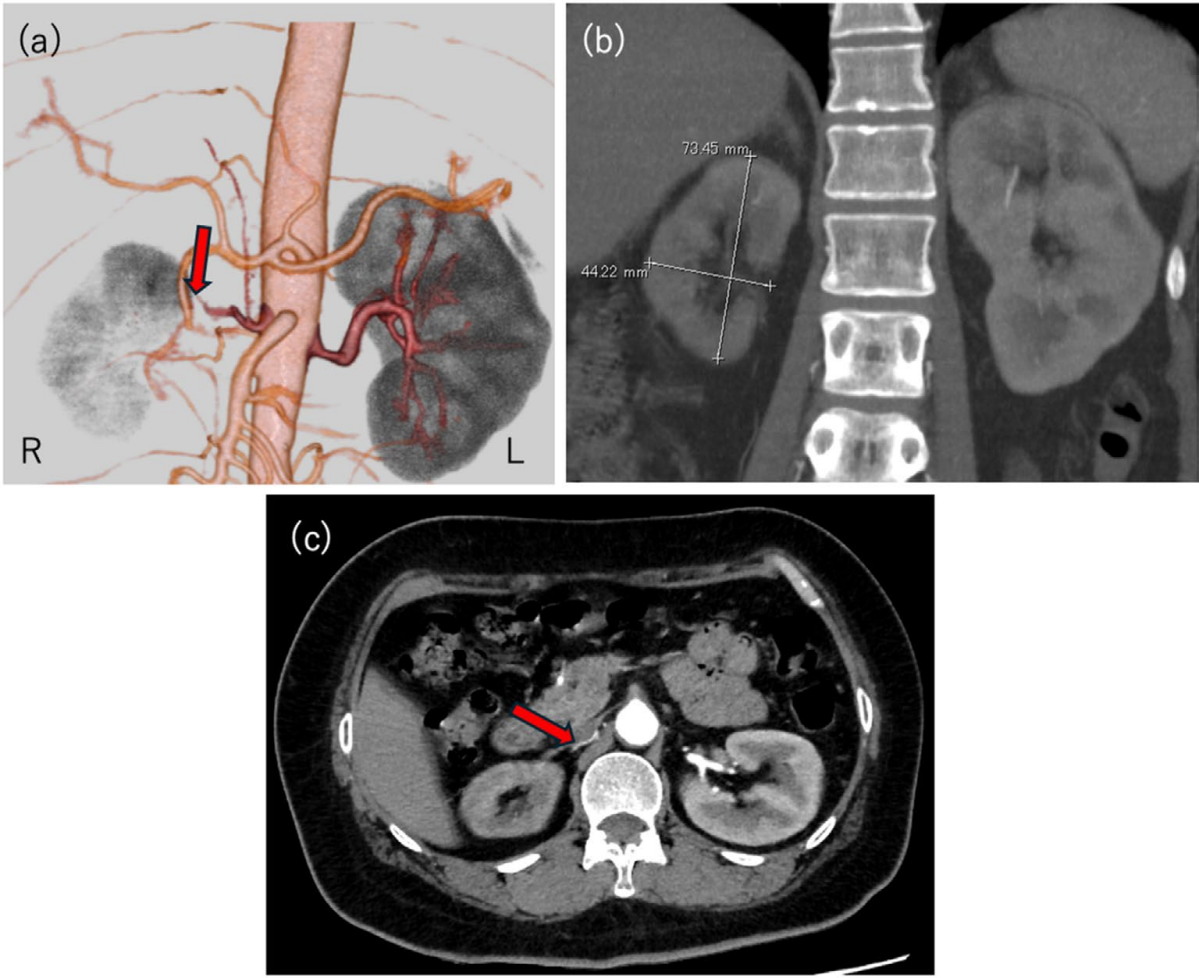
(a) 3D reconstruction of computed tomography (CT) images. Stenosis is observed in the right renal artery (arrow). (b) Contrast‐enhanced CT (coronal section). Right kidney atrophy of 7.3 cm by 4.4 cm. (c) Contrast‐enhanced CT (axial section). Stenosis is observed in the right renal artery (arrow).

A 49‐year‐old woman with no significant medical history presented with progressive fatigue, muscle weakness, and difficulty walking. Her blood pressure was 172/126 mmHg. Laboratory tests revealed hypokalemia (K: 2.3 mEq/L), elevated serum creatinine (Cr: 1.12 mg/dL), and reduced estimated glomerular filtration rate (eGFR: 41.4 mL/min/1.73 m^2^). She was admitted to the nephrology department for further evaluation. Endocrinological tests showed markedly elevated plasma renin activity (153 ng/mL/h) and plasma aldosterone concentration (246 pg/mL). The captopril challenge test yielded an aldosterone‐to‐renin ratio of 0.71, which did not support a diagnosis of primary aldosteronism. Hypertension due to high renin levels was suspected (Table 1). Contrast‐enhanced computed tomography (CT) revealed severe right renal atrophy (7.3 × 4.4 cm) and significant stenosis of the right main renal artery. The left kidney showed compensatory hypertrophy (10.1 × 7.0 cm), with mild stenosis of the proximal left renal artery (Figure 1).

**TABLE 1:** Blood test after admission.

				Reference values
	TSH	1.74	(μIU/mL)	(0.38–4.31)
	FT3	1.4	(pg/mL)	(2.1–3.1)
	FT4	0.73	(ng/dL)	(0.75–1.42)
	Plasma renin activity	153	(ng/mL/h)	(0.2–2.3)
	Plasma aldosterone concentration	246	(pg/mL)	(4.0–82.1)
	Cortisol	17.5	(μg/dL)	(4.4–21.1)
	ACTH	9.8	(pg/mL)	(8.7–61.5)
	Adrenaline	16	(pg/mL)	(~100)
	Noradrenaline	347	(pg/mL)	(100–450)
	Dopamine	8	(pg/mL)	(~20)
After the captopril challenge test	Plasma renin activity	229	(ng/mL/h)	(0.2–2.3)
	Plasma aldosterone concentration	162	(pg/mL)	(4.0–82.1)
	Aldosterone/renin	0.71		~200

The patient was diagnosed with RVH due to bilateral renal artery stenosis, with right‐sided dominance. Initial blood pressure control was achieved with carvedilol, nifedipine, and amlodipine, and the patient was discharged on day 15. Despite ongoing antihypertensive management during outpatient follow‐up, the patient's blood pressure progressively deteriorated, necessitating an increase in both the number and dosage of antihypertensive medications. Nine months after discharge, the patient developed dyspnea and was readmitted due to acute heart failure secondary to RVH. Emergency dialysis was performed for volume removal. The antihypertensive regimen was intensified to a five‐drug regimen. The patient was subsequently discharged. As the patient was considered to have a high risk of heart failure recurrence with pharmacological treatment alone, additional evaluations were conducted to explore further therapeutic interventions for RVH.

Renal vein sampling demonstrated approximately fourfold higher renin secretion from the right renal vein than the left, confirming right‐sided dominance in RVH. The time‐activity curve of the 99mTc‐MAG3 renogram demonstrated a pattern consistent with severe functional impairment of the right kidney. The standardized effective renal plasma flow was 8.0 mL/min in the right kidney and 28.4 mL/min in the left kidney, indicating a marked reduction of right renal function.

At our institution's cardiology department, PTRA was considered for the treatment of right RAS. However, several factors led to the recommendation of right nephrectomy instead: (1) 99mTc‐MAG3 scintigraphy demonstrated significantly reduced function of the right kidney, suggesting that renal preservation would offer minimal benefit; (2) severe stenosis of the right renal artery, which was expected to make endovascular intervention technically challenging; and (3) the high restenosis rate and poor long‐term outcomes associated with PTRA. Following consultation with a cardiologist, PTRA was considered to offer limited therapeutic benefit. Surgical resection was recommended, and the patient was referred to our department. Two months after the consultation, laparoscopic right nephrectomy was performed. Intraoperatively, the renal artery appeared unusually firm and rigid, suggestive of underlying atherosclerotic changes.

**FIGURE 2:**
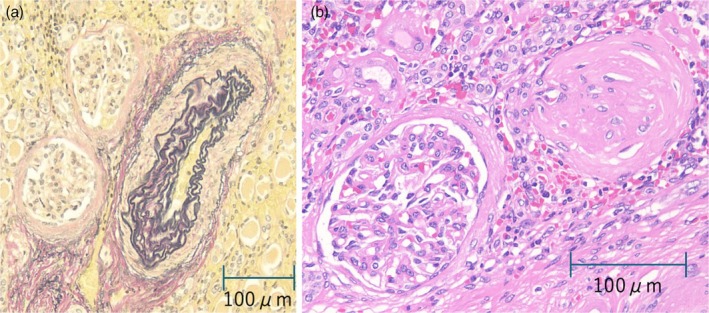
(a) Partial stenosis of the right renal artery. (b) Focal glomerular sclerosis of the right kidney.

Histopathological examination of the excised kidney revealed segmental arterial stenosis and partial glomerular hyalinization (Figure 2). Thus, atherosclerotic RAS was suspected as the underlying cause of the right renal artery stenosis.

**FIGURE 3:**
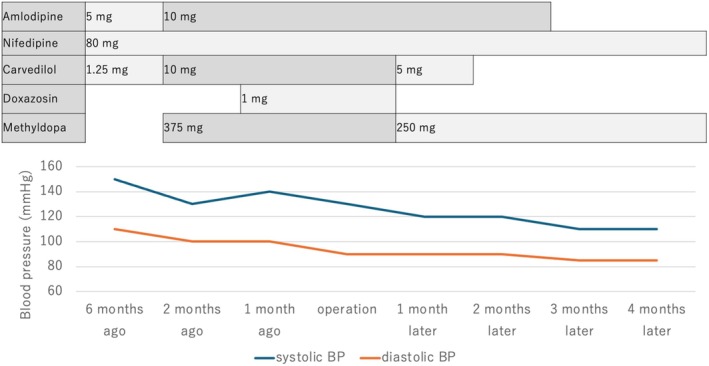
Pre‐ and postoperative blood pressure and medication changes.

Postoperatively, plasma renin activity and aldosterone concentration declined significantly. The patient's antihypertensive regimen was tapered, with discontinuation of doxazosin on postoperative day (POD) 2, and reduction of carvedilol from 10 to 5 mg/day and methyldopa from 375 to 250 mg/day by POD 7. We monitored renal function through regular blood tests in collaboration with the nephrology department. Follow‐up imaging studies, including ultrasound and contrast‐enhanced CT, were performed every few months, and blood tests were conducted monthly. At 4 months postoperatively, blood pressure remained well controlled using nifedipine and methyldopa (Figure 3). However, serum creatinine had increased to 2.9 mg/dL (preoperative level: 2.5 mg/dL), and eGFR had declined to 14.6 mL/min/1.73 m^2^ (preoperative level: 17.0 mL/min/1.73 m^2^), indicating a mild deterioration in renal function. Nevertheless, the patient remained dialysis independent throughout the follow‐up period (Figure S1).

## 3 Discussion

The treatment of RVH associated with RAS typically involves a combination of RAA inhibitors, CCBs, and beta‐blockers. However, in bilateral RAS, ACE inhibitors may worsen renal function [5]. In this case, the patient was initially managed with CCBs and beta‐blockers; however, blood pressure control remained challenging. Administration of RAA inhibitors was considered; however, contrast‐enhanced CT revealed bilateral RAS. Therefore, we administered an alpha‐blocker, and blood pressure was managed with a total of five antihypertensive agents.

For drug‐resistant RVH, PTRA is a potential treatment option. However, PTRA has a restenosis rate of 14%–57%, and long‐term outcomes are suboptimal in some cases [6]. Moreover, if the renal size is less than 8 cm, the likelihood of improved blood pressure or renal function recovery following PTRA or surgical revascularization is minimal [7]. In cases where renal size and function fail to improve after PTRA, no significant antihypertensive effect is achieved [8]. Previous studies have shown that revascularization success depends on factors such as collateral circulation, preserved glomeruli, renal length ≥ 9 cm, and functional imaging findings [9, 10]. In this case, contrast‐enhanced CT showed the affected kidney was atrophic, measuring 7.3 cm in length, and renography indicated severe functional impairment. Consultation with a cardiologist confirmed that the RAS was too severe to be visualized on contrast‐enhanced CT. Thus, PTRA would be technically difficult. After a thorough discussion with the patient, laparoscopic right nephrectomy was selected as the treatment strategy.

Nephrectomy has been reported to be an effective treatment for RVH in cases where blood pressure cannot be controlled with medication; PTRA is unfeasible, and the affected kidney is atrophic (< 8 cm) with impaired renal function [11]. The present case met all these criteria. The primary disadvantage of nephrectomy is the risk of deterioration of renal function due to the resulting solitary kidney. In the present case, the contralateral kidney also exhibited stenotic changes, likely due to atherosclerosis, suggesting the potential for disease progression. Careful long‐term follow‐up was necessary.

## 4 Conclusion

This report describes a case of drug‐resistant RVH caused by severe RAS that was successfully managed with nephrectomy of the affected kidney.

## Consent

Written informed consent was given by the patient for the publication of this article and the accompanying images.

## Conflicts of Interest

The authors declare no conflicts of interest.

## Supporting information


**Data S1:** iju570084‐sup‐0001‐DataS1.docx.
