# Supplementary figures and images for: A Case of Drug‐Resistant Renovascular Hypertension due to Renal Artery Stenosis Successfully Treated by Nephrectomy of the Affected Kidney
Source: IJU Case Rep. 2025 Aug 19;8(5):525–8. doi: 10.1002/iju5.70084 (PMC12408181; doi:10.1002/iju5.70084)

1. List of supporting information

Supplementary: Postoperative course of renal function


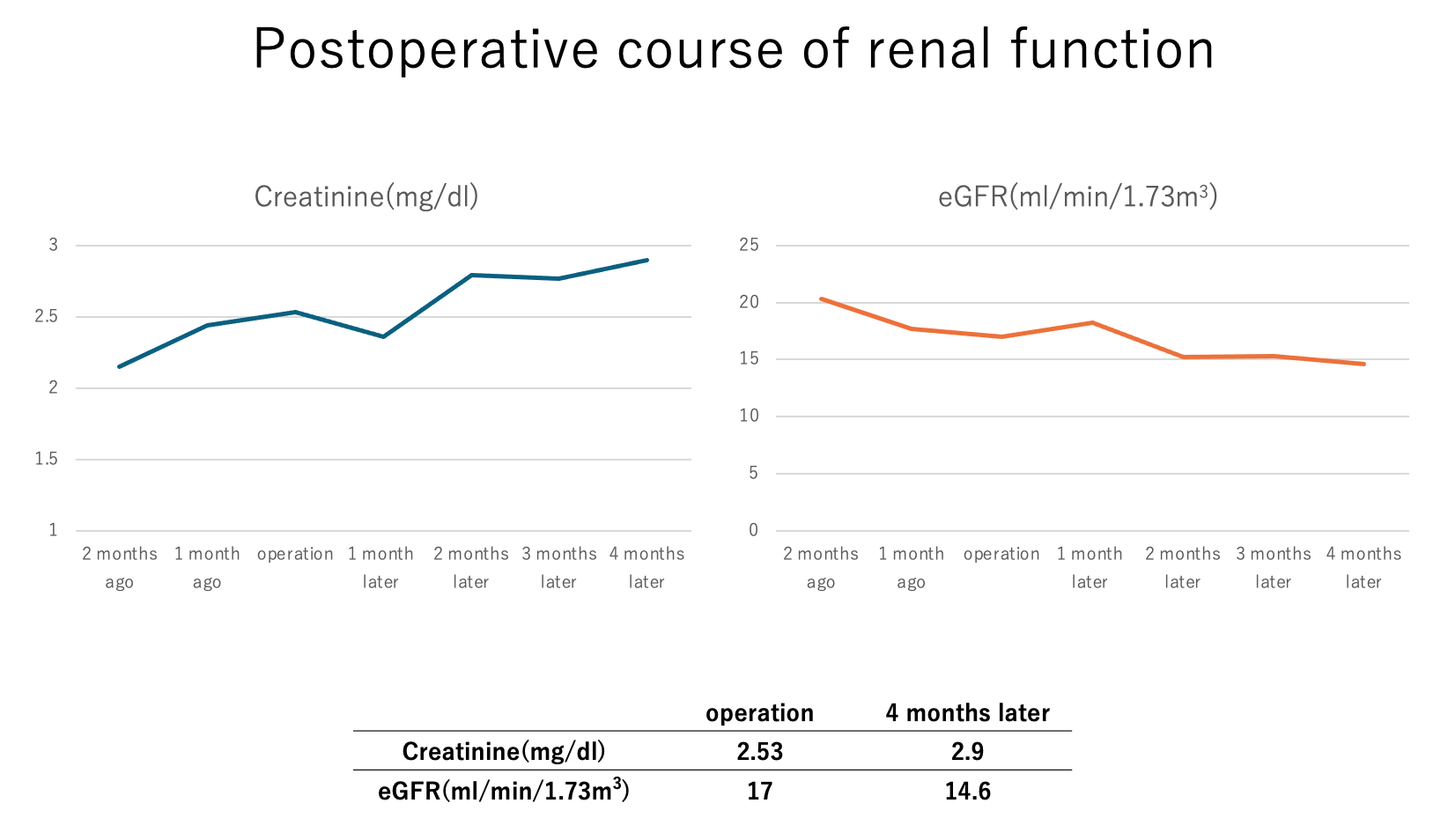

Supplement: Supplementary file 1 — Data S1: iju570084‐sup‐0001‐DataS1.docx. [file IJU5-8-525-s001.docx]
